# Surgical treatment outcomes of acetabular posterior wall and posterior column fractures using 3D printing technology and individualized custom-made metal plates: a retrospective study

**DOI:** 10.1186/s12893-024-02451-x

**Published:** 2024-05-16

**Authors:** He Zhang, Hong-Peng Guo, Rong-Da Xu, Si-Yu Duan, Hai-Rui Liang, Zhen-Cun Cai

**Affiliations:** 1 Department of Orthopaedic Surgery, Central Hospital Affiliated to Shenyang Medical College, Shenyang, Liaoning China; 2 Department of General Surgery, The Center Hospital of Shenyang Sujiatun, Shenyang, Liaoning China; 3 Key Laboratory of Human Ethnic Specificity and Phenomics of Critical Illness in Liaoning Province, Shenyang Medical College, Shenyang, Liaoning China

**Keywords:** 3D printing, Individualized custom-made metal plates, Acetabular fractures, Internal fixation surgery

## Abstract

**Background:**

Fractures involving the posterior acetabulum with its rich vascular and neural supply present challenges in trauma orthopedics. This study evaluates the effectiveness of 3D printing technology with the use of custom-made metal plates in the treatment of posterior wall and column acetabular fractures.

**Methods:**

A retrospective analysis included 31 patients undergoing surgical fixation for posterior wall and column fractures of the acetabulum (16 in the 3D printing group, utilizing 3D printing for a 1:1 pelvic model and custom-made plates based on preoperative simulation; 15 in the traditional group, using conventional methods). Surgical and instrument operation times, intraoperative fluoroscopy frequency, intraoperative blood loss, fracture reduction quality, fracture healing time, preoperative and 12-month postoperative pain scores (Numeric Rating Scale, NRS), hip joint function at 6 and 12 months (Harris scores), and complications were compared.

**Results:**

The surgical and instrument operation times were significantly shorter in the 3D printing group (*p* < 0.001). The 3D printing group exhibited significantly lower intraoperative fluoroscopy frequency and blood loss (*p* = 0.001 and *p* < 0.001, respectively). No significant differences were observed between the two groups in terms of fracture reduction quality, fracture healing time, preoperative pain scores (NRS scores), and 6-month hip joint function (Harris scores) (*p* > 0.05). However, at 12 months, hip joint function and pain scores were significantly better in the 3D printing group (*p* < 0.05). Although the incidence of complications was lower in the 3D printing group (18.8% vs. 33.3%), the difference did not reach statistical significance (*p* = 0.433).

**Conclusion:**

Combining 3D printing with individualized custom-made metal plates for acetabular posterior wall and column fractures reduces surgery and instrument time, minimizes intraoperative procedures and blood loss, enhancing long-term hip joint function recovery.

**Clinical Trial Registration:**

12/04/2023;Trial Registration No. ChiCTR2300070438; http://www.chictr.org.cn.

**Supplementary Information:**

The online version contains supplementary material available at 10.1186/s12893-024-02451-x.

## Introduction

The anatomical structure of acetabular fractures is exceptionally complex, particularly in cases involving fractures of the posterior wall and column. Due to the abundant vascular and neural supply in the posterior acetabulum, surgical procedures pose significant risks and challenges, even for experienced orthopedic surgeons [1, 2]. Surgical treatment is commonly employed for posterior wall and column fractures of the acetabulum, with anatomical reduction being a crucial determinant for achieving favorable postoperative functional recovery [3, 4]. Traditional surgical treatment of complex acetabular fractures involves assessing the fracture type based on the physician’s experience and imaging data, and then formulating surgical plans. During surgery, doctors often temporarily bend conventional anatomical plates to better match the reduced position of the acetabular fracture. This process involves significant surgical risks and challenges. With the advancement of digital orthopedic technology, 3D printing has found increasing application in the clinical treatment of complex fractures in recent years. Physicians can intuitively understand the extent and type of fracture injury through 3D models, facilitating preoperative surgical planning and aiding in comparing intraoperative fracture reduction, with some positive outcomes [5–7].

However, based on the literature search, it was found that the current application of 3D printing technology in the treatment of complex fractures mainly involves printing models for accurate diagnosis and surgical planning. Precontoured anatomic plates designed based on the fracture model are sterilized and prepared for surgery [8]. During surgery, traditional anatomical plates are still used for fixation. If the plates do not fit properly, they need to be re-bent, which further reduces the strength of the plates and affects stable fixation after fracture reduction [9].

To address this issue, our research team used computer-simulated fracture reduction to design the placement, shape, and size of metal plates based on the virtually reduced fracture model. We utilized 3D printing technology to create individualized custom-made metal plates required during surgery. This approach eliminates the need for intraoperative plate bending, effectively stabilizes the fracture ends, and further reduces the complexity of the surgery. In this study, we conducted a retrospective comparison of surgical outcomes between using 3D printing technology with individualized custom-made metal plates and traditional surgical treatment for posterior wall and column fractures of the acetabulum.

## Materials and methods

This study retrospectively evaluated 95 patients with acetabular fractures who underwent surgical treatment at the Affiliated Central Hospital of Shenyang Medical College from May 2019 to May 2022. Given that the individualized custom-made metal plates used in this study were specifically designed for the treatment of posterior wall and column acetabular fractures, the inclusion criteria were as follows: (1) age ≥ 18 years; (2) diagnosed with posterior wall and column acetabular fractures based on the Letournel-Judet classification; (3) underwent open reduction and internal fixation surgery; (4) complete follow-up data. Exclusion criteria included: (1) old fractures; (2) Letournel-Judet fractures of other types; (3) concomitant sciatic nerve injury; (4) lower limb immobility due to other reasons before injury. Based on the inclusion and exclusion criteria, a total of 31 patients were included in this study.

All patients underwent preoperative pelvic anteroposterior X-ray examination (Netherlands, Philips digital radiography DR system) and CT scanning with three-dimensional reconstruction of the affected hip (Netherlands, Philips 256-row spiral CT machine, scanning layer thickness 0.6 mm). Based on the surgical treatment received by the patients, the 31 patients with posterior wall and column fractures of the acetabulum were divided into two groups: the 3D printing group (16 cases) and the traditional group (15 cases). This study has been approved by the Medical Ethics Committee of Shenyang Medical College Affiliated Central Hospital (Approval No. 2,022,018). All patients provided verbal or written informed consent. The study has been registered at https://www.chictr.org.cn/.

### 3D Printing model and individualized custom-made metal plates

**Fig. 1:**
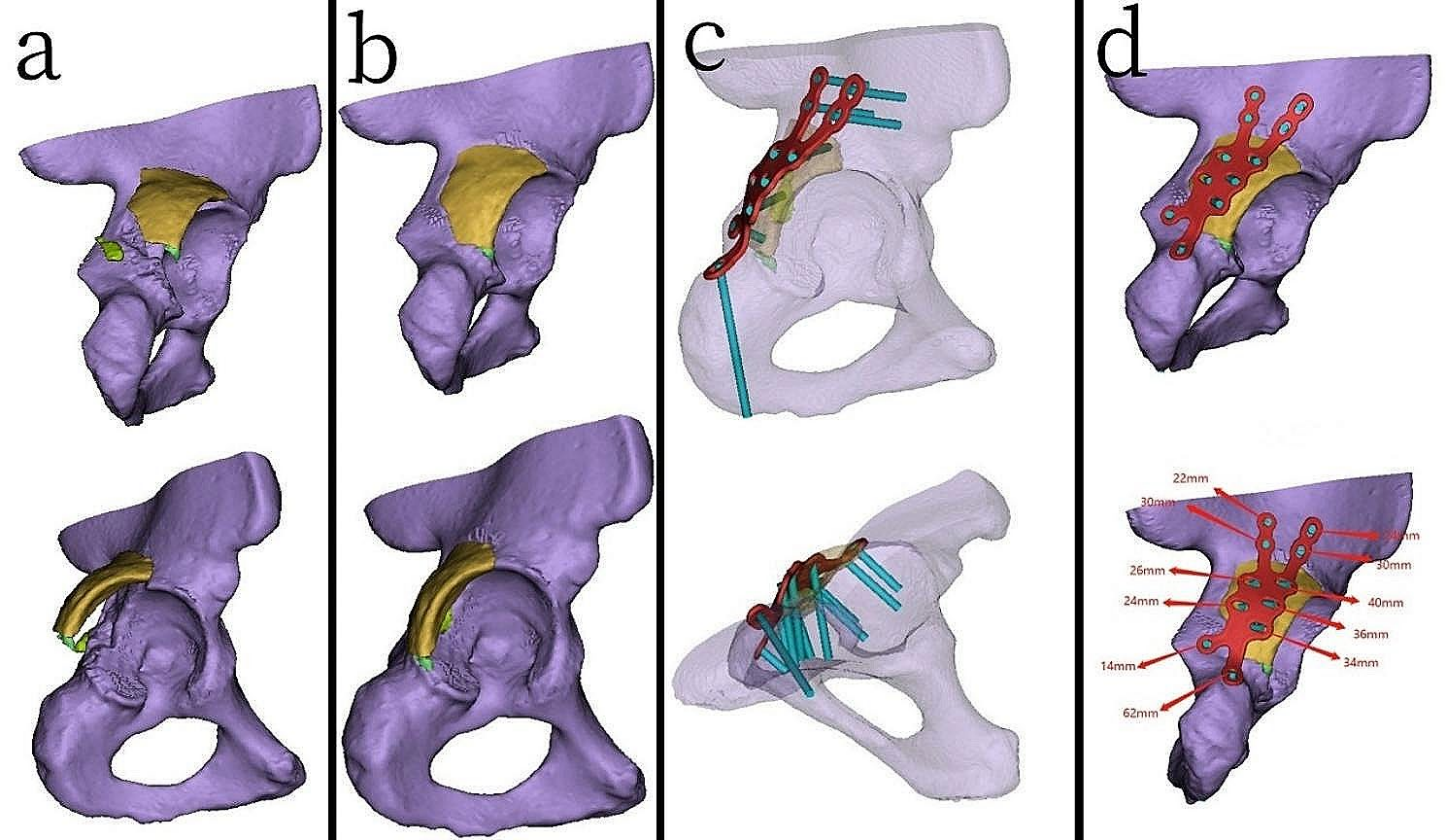
Computer-aided three-dimensional modeling of fracture patterns and the production of individualized custom-made metal plates (**a**) Three-dimensional fracture model simulated using Mimics software (**b**) Three-dimensional fracture model after computerized virtual reduction (**c**) Confirmation of the screw directions and lengths for individualized custom-made metal plates, ensuring that the implanted screws will not enter the joint cavity (**d**) Positioning of individualized custom-made metal plates on the virtually reduced fracture model, along with the recommended lengths for each screw on the metal plates

In the 3D printing group, the CT imaging data of the affected side was imported into Mimics 20.0 software (Materialise, Belgium) in DICOM format. The femur and spine regions were removed using the delete function, and a three-dimensional model of the acetabular fracture was created (Fig. 1a). Based on the modeled acetabular fracture, the fracture fragments were separated using the threshold circle selection function, and virtual anatomical reduction of the fracture was performed using the move and rotate functions (Fig. 1b). Subsequently, the three-dimensional modeling data of the fracture and the virtually reduced fracture were exported in STL format and imported into FlashPrint 5 software (Shanzhu Technology, China) for 3D printing. Polylactic acid (PLA) was used as the printing material to produce physical models of the fracture and the virtually reduced fracture.

Using the physical model of the virtually reduced fracture, an engineer (holding an intermediate professional title specializing in medical device engineering) collaborated with the surgical team to customize an individualized metal plates. The optimal position for placing the metal plates was determined according to the type of fracture, achieving stable reduction of the fracture area. The customization process included designing the shape of the metal plates, the fixation area, and the positions and orientations of the screw holes. The thickness of the individualized custom-made metal plates was determined based on the location of the fracture, typically ranging from 3 to 4 mm.

**Fig. 2:**
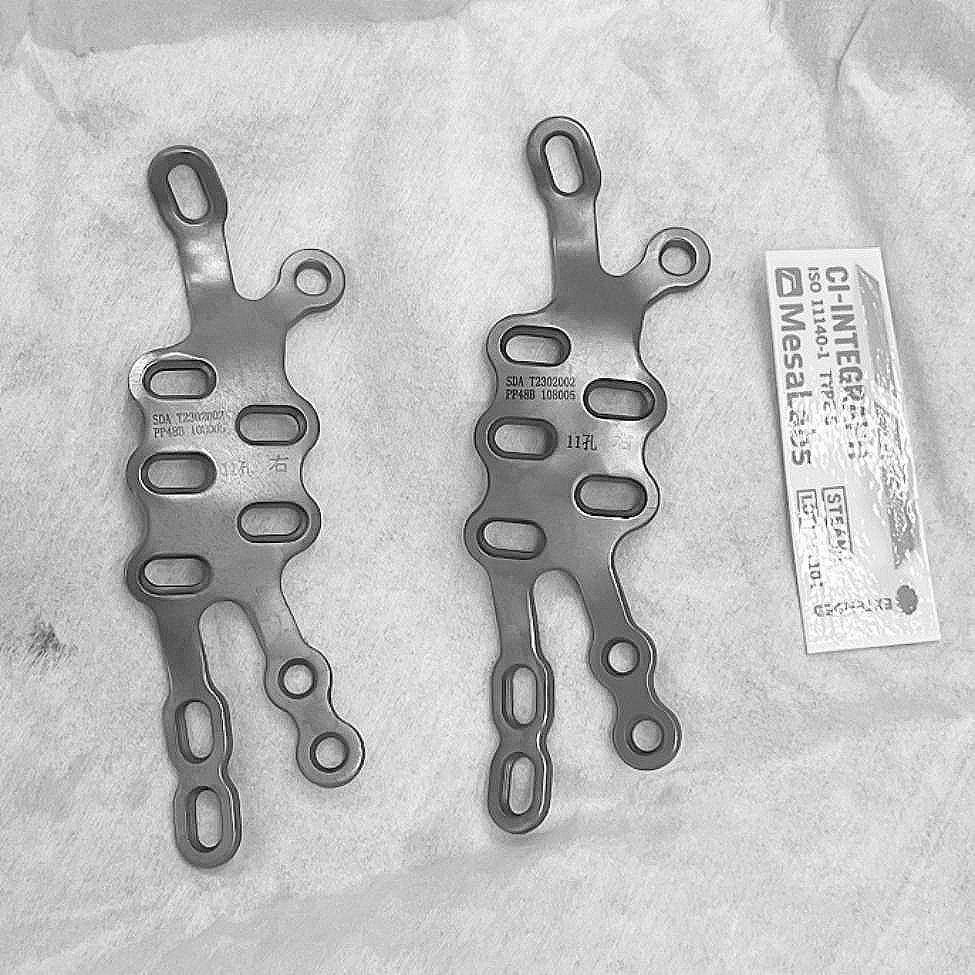
Pre-operatively, the individualized custom-made metal plates, which have undergone processing and passed quality inspection, are disinfected and ready for use

An engineer utilizes Unigraphics NX software (Siemens PLM Software, USA) for detailed design of the metal plates. Subsequently, using PLA as the printing material, the physical models of the metal plates were printed using FlashPrint 5 software. The physical models of the metal plates were then imported into Mimics software through reverse scanning. On the virtually reduced fracture model, the metal plates were virtually placed, and the diameter and direction of the screws were confirmed. Transparency was applied to the fracture model to ensure that the implanted screws would not enter the joint cavity (Fig. 1c). The length of the screws was measured, and the recommended lengths for each screw hole on the metal plates were marked (Fig. 1d). Based on the production drawings of the custom-made metal plates, pure titanium TA3 was used as the raw material for machining and shaping the metal plates at the processing plant, resulting in the creation of actual individualized custom-made metal plates. The metal plates then underwent additional processes such as sandblasting, magnetic polishing, and ultrasonic cleaning before being sent to a specialized quality inspection department for testing. The quality inspection included assessments of material hardness, bending strength, equivalent bending stiffness, surface roughness, dimensional accuracy, and the fitting performance between the screws and the metal plates. After passing the tests, the metal plates were marked and packaged. Prior to the surgery, the metal plates were subjected to high-temperature and high-pressure disinfection and sterilization( Fig. 2).

### Surgical technique

**Fig. 3:**
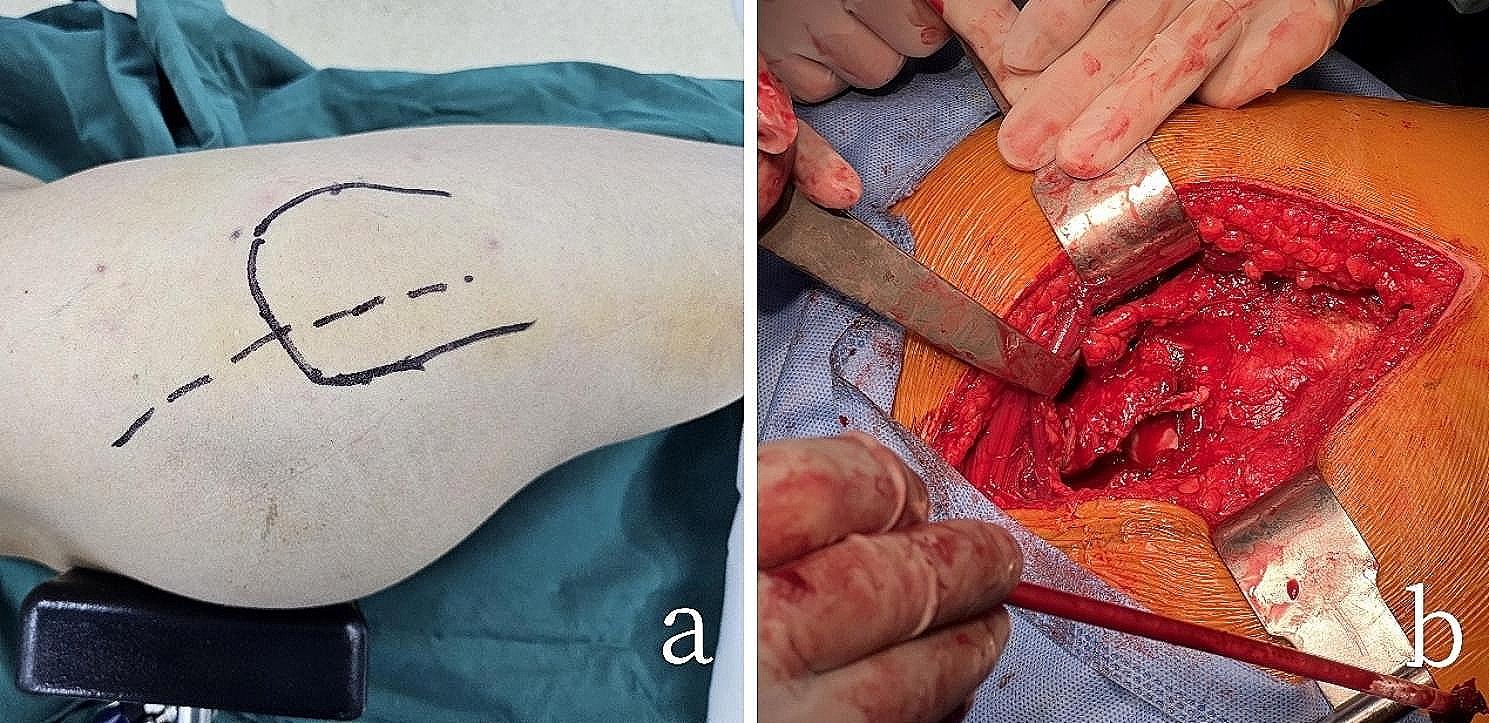
The patient’s positioning and surgical approach during the operation (**a**) Surgical incision using the Kocher-Langenbeck approach (**b**)Intraoperative view of the exposed acetabular posterior wall and column fractures

All surgeries were performed by an experienced trauma orthopedic surgeon. After the patient was placed under general anesthesia, they were positioned in the lateral decubitus position, and the hip area and the affected limb were disinfected with iodine. Sterile drapes were used to cover the area. Both groups of patients underwent the Kocher-Langenbeck approach, with an incision starting from the posterior superior iliac spine and curving downward over the apex of the greater trochanter of the femur, extending vertically for approximately 15 cm. During the surgery, the incision could be extended as needed to adequately expose the acetabular posterior wall and column fractures (Fig. 3).

**Fig. 4:**
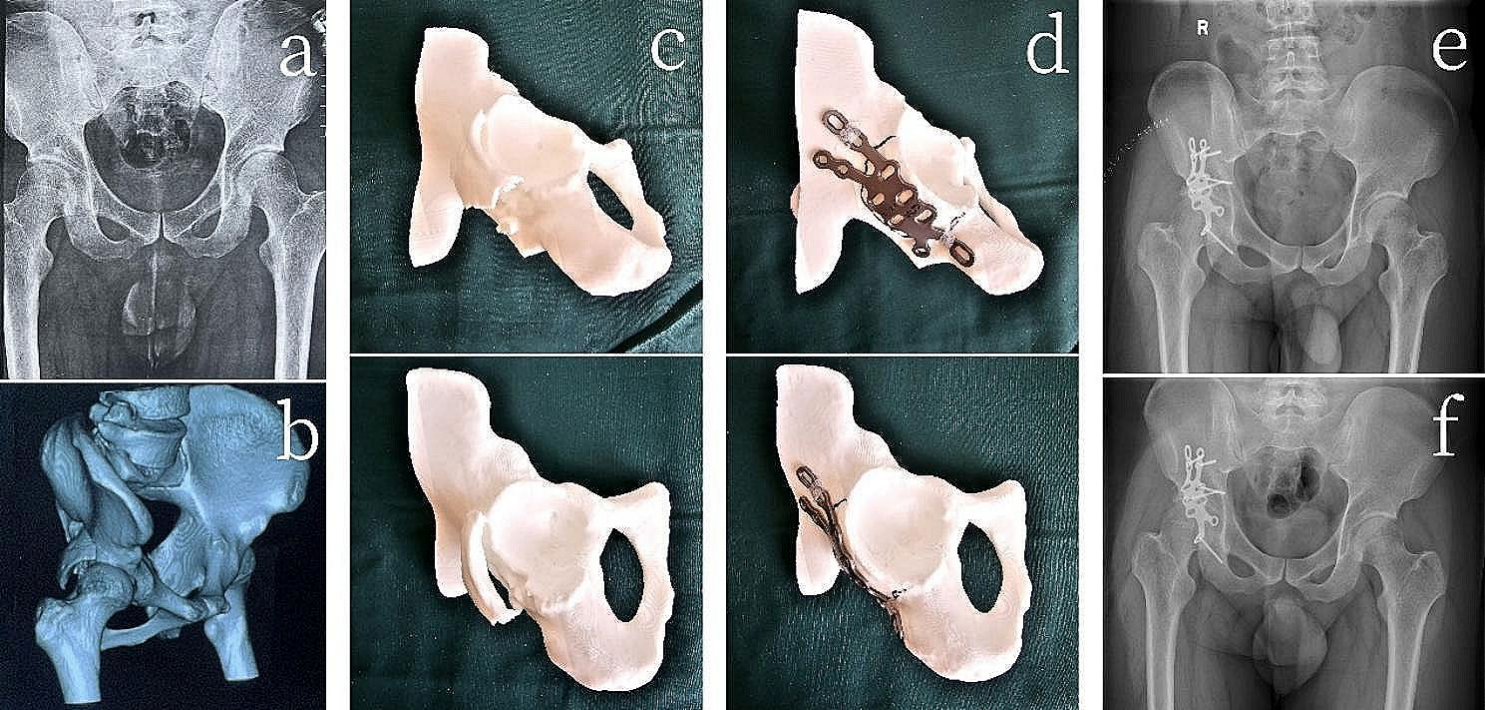
A 21-year-old patient with a right acetabular posterior wall and column fracture (3D printing group) (**a**) Preoperative X-ray (anteroposterior view) (**b**) three-dimensional reconstructed computed tomography (CT) images (**c**) 3D-printed model of the fracture (anteroposterior and lateral views) (**d**) 3D-printed models after computer virtual reduction and customized individualized metal plate fitting (anteroposterior and lateral views) (**e**) Postoperative day one pelvic X-ray (anteroposterior view) (**f**) Follow-up pelvic X-ray at 3 months postoperatively (anteroposterior view)

In the 3D printing group, the fractures were reduced based on the preoperative 3D physical models, and individualized custom-made metal plates, designed and fabricated beforehand, were applied. The metal plates were fixed in place by adhering to the pre-designed bony landmarks according to the model (Fig. 4). In the traditional surgery group, the fractures were reduced based on the surgeon’s experience, and conventional metal plates were contoured and fixed based on the reduced fractures. In both the 3D printing group and the traditional surgery group, the lengths of the screws were measured and implanted at appropriate positions. Intraoperative fluoroscopy was used to assess the reduction of the fractures, determine the positioning of the metal plates, ensure that the screws did not penetrate into the acetabulum, and conduct multidirectional mobility tests of the affected hip joint. The stability of the fracture fragments was observed to ensure the success of the surgery.

### Postoperative management

On the first day after surgery, both groups of patients underwent pelvic anteroposterior X-ray examination. During hospitalization, routine analgesic medication and subcutaneous injections of anticoagulants were administered. After discharge, oral anticoagulants were prescribed for 2 weeks to prevent deep venous thrombosis in the lower limbs. Follow-up visits were scheduled every month during the postoperative period, and pelvic anteroposterior X-ray images were obtained, with a minimum follow-up time of 12 months. On the day of the surgery, active rehabilitation exercises were initiated for the affected side hip joint, followed by partial weight-bearing exercises after 6 weeks. At 3 months postoperatively, full weight-bearing exercises were allowed based on the X-ray findings.

### Evaluation indicators

Compare the data between the two groups, including patient age, gender, BMI, length of hospital stay, time from injury to surgery, and average follow-up time.The surgical time and instrument operation time were recorded for both groups. Surgical time was defined as the time from skin incision to suture completion, while instrument operation time was defined as the time from the beginning of adjusting and placing the metal plate to the insertion of the last screw. The 3D printing model time referred to the time from design to the printing of the physical model of the fracture. The custom-made metal plate production time was defined as the time from design to the processing of the individualized custom-made metal plate. Intraoperative blood loss and the number of intraoperative fluoroscopy procedures were also recorded. Intraoperative blood loss was calculated by measuring the volume of flushing fluid, blood in the suction bottle, and blood in the gauze. The number of intraoperative fluoroscopy procedures referred to the total number of times fluoroscopy was performed during the surgery. Radiological evaluations were conducted by two experienced orthopedic surgeons, and the radiological results were scored based on the maximum residual displacement of the postoperative fracture: fracture reduction quality was classified as good (0–2 mm displacement) or fair (≥ 2 mm displacement). Both groups of patients utilized the Numeric Rating Scale (NRS) to assess fracture pain preoperatively, employing a scale from 0 (“no pain”) to 10 (“unbearable fracture pain”), where each integer denoted one unit of pain intensity. Subsequently, 12 months after the operation, patients underwent follow-up assessments for NRS pain scoring. At 6 and 12 months postoperatively, hip joint function was evaluated using the Harris scoring system [10]: hip joint function was considered excellent/good (Harris score ≥ 80 points) or fair/poor (Harris score < 80 points). Complications included inflammatory reactions, heterotopic ossification, infection, nonunion of the fracture, iatrogenic nerve symptoms, and traumatic arthritis.

### Statistical analysis

Statistical analysis was performed using SPSS software (version 26.0, USA). Categorical variables were presented as numbers or percentages, while continuous variables were presented as mean ± standard deviation. Fisher’s exact test was used to assess categorical variables. The Shapiro-Wilk test was used to assess whether continuous variables followed a normal distribution. For normally distributed continuous variables, such as age, BMI, time from injury to surgery, instrument operation time, and fracture healing time, independent sample t-tests were used for analysis. For non-normally distributed continuous variables, such as length of hospital stay, average follow-up time, surgical time, number of X-ray fluoroscopy procedures, and intraoperative blood loss, Mann-Whitney U tests were used for analysis. The significance level for all statistical tests was set at *p* < 0.05.

## Results

### Demographic and clinical data

This study included a total of 31 patients, with 16 patients in the 3D printing group (13 males and 3 females), with a mean age of 35.2 ± 9.5 years and a mean BMI of 25.0 ± 3.1 kg/m². The traditional group comprised 15 patients (10 males and 5 females) with a mean age of 37.3 ± 10.8 years and a mean BMI of 24.5 ± 2.4 kg/m². Demographic characteristics between the two groups were similar (all *p* > 0.05). The length of hospital stay in the 3D printing group (mean 11.6 ± 1.7 days) was slightly longer than that in the traditional group (mean 10.9 ± 1.8 days), but the difference was not statistically significant (*p* = 0.276). The time from injury to surgery in the 3D printing group (mean 154.3 ± 21.5 h) was longer than that in the traditional group (mean 142.8 ± 20.9 h), but the difference was not statistically significant (*p* = 0.146). The average follow-up time for both groups exceeded 12 months (Table 1).

### Surgical data

In the 3D printing group, the average time required to print the physical model was 15.9 ± 3.5 h, and the average time to produce the individualized custom-made metal plates was 32.9 ± 6.3 h. The 3D printing group had shorter average surgical time, instrument operation time, X-ray fluoroscopy times, and intraoperative blood loss compared to the traditional group (X-ray fluoroscopy times *p* = 0.001, all other indicators *p* < 0.001). Specifically, the average surgical time in the 3D printing group was (110.0 ± 12.6 min), compared to (166.0 ± 6.4 min) in the traditional group; the average instrument operation time in the 3D printing group was (34.2 ± 6.2 min), compared to (58.1 ± 8.6 min) in the traditional group; the average X-ray fluoroscopy times in the 3D printing group was (6 ± 2 times), compared to (8 ± 2 times) in the traditional group; the average intraoperative blood loss in the 3D printing group was (254.9 ± 57.2 mL), compared to (401.6 ± 46.6 mL) in the traditional group (Table 2).

### Postoperative functional and radiological data

The postoperative fracture reduction quality in the 3D printing group (good rate: 15/16, 93.8%) was numerically higher than in the traditional group (good rate: 12/15, 80%), but the difference was not statistically significant (*p* = 0.333). The average healing time in the 3D printing group, 14.2 ± 1.1 weeks, was numerically shorter than in the traditional group, 14.8 ± 1.4 weeks, but the difference was not statistically significant (*p* = 0.215). The preoperative NRS score in the 3D printing group was 8.0 ± 1.1, which did not significantly differ from the preoperative NRS score in the traditional group (7.5 ± 0.8) (*p* = 0.153). However, the 12-month NRS score in the 3D printing group (0.8 ± 0.8) was significantly lower than that in the traditional group (1.7 ± 1.0), with a statistically significant difference (*p* = 0.017). The 6-month hip joint function (Harris score) in the 3D printing group (excellent/good rate: 14/16, 87.5%) was numerically higher than in the traditional group (excellent/good rate:11/15, 73.3%), but the difference was not statistically significant (*p* = 0.394). However, the 12-month hip joint function (Harris score) in the 3D printing group (excellent/good rate:15/16, 93.8%) was significantly better than in the traditional group (excellent/good rate:9/15, 60.0%), and the difference was statistically significant (*p* = 0.037) (Table 3).

### Complications

The incidence of complications was similar between the two groups (*p* = 0.433), and no cases of bone infection or nonunion were observed in either group. In the 3D printing group (complication rate: 3/16, 18.8%), two patients experienced inflammatory reactions, and one patient developed heterotopic ossification one month after surgery. In the traditional group (complication rate: 5/15, 33.3%), two patients experienced inflammatory reactions, one patient developed heterotopic ossification two months after surgery, one patient developed traumatic arthritis, and one patient experienced iatrogenic nerve symptoms, which resolved six months after surgery (Table 3).

**Table 1:** Patient demographics and clinical data

	3D Printing Group (*n* = 16)	Traditional Group (*n* = 15)	*p* value
Age (years)	35.2 ± 9.5	37.3 ± 10.8	0.574
Sex, n (%)			0.433
Male	13 (81.3)	10 (66.7)	
Female	3 (18.8)	5 (33.3)	
BMI (kg/m²)	25.0 ± 3.1	24.5 ± 2.4	0.617
Length of Hospital Stay (days)	11.6 ± 1.7	10.9 ± 1.8	0.276
Time from Injury to Surgery (hours)	154.3 ± 21.5	142.8 ± 20.9	0.146
Average Follow-up Time (months)	14.0 ± 1.5	14.3 ± 2.0	0.812

**Table 2:** Perioperative clinical characteristics of patients

	3D Printing Group (*n* = 16)	Traditional Group (*n* = 15)	*p* value	
3D Printing Model Time (hours)	15.9 ± 3.5	-		-
Customized Plate Production Time (hours)	32.9 ± 6.3	-		-
Average Surgical Time (minutes)	110.0 ± 12.6	166.0 ± 6.4		< 0.001
Instrument Operation Time (minutes)	34.2 ± 6.2	58.1 ± 8.6		< 0.001
X-ray Fluoroscopy (times)	6 ± 2	8 ± 2		0.001
Intraoperative Blood Loss (mL)	254.9 ± 57.2	401.6 ± 46.6		< 0.001

**Table 3:** Patient surgical outcome indicators

	3D Printing Group (*n* = 16)	Traditional Group (*n* = 15)	*p* value
Fracture Reduction Quality, n (%)			0.333
Good (0–2 mm)	15 (93.8)	12 (80.0)	
Fair (> 2 mm)	1 (6.3)	3 (20.0)	
Fracture Healing Time (weeks)	14.2 ± 1.1	14.8 ± 1.4	0.215
Preoperative NRS (score)	8.0 ± 1.1	7.5 ± 0.8	0.153
12-month NRS (score)	0.8 ± 0.8	1.7 ± 1.0	0.017
6-month Harris Score, n (%)			0.394
Excellent/Good (≥ 80)	14 (87.5)	11 (73.3)	
Fair/Poor (< 80)	2 (12.5)	4 (26.7)	
12-month Harris Score, n (%)			0.037
Excellent/Good (≥ 80)	15(87.5)	9 (73.3)	
Fair/Poor (< 80)	1 (12.5)	6 (26.7)	
Complications, n (%)			0.433
None	13 (81.3)	10 (66.7)	
Present	3 (18.8)	5 (33.3)	

## Discussion

Posterior column acetabular fractures are a relatively common type of acetabular fracture and are typically treated using the Kocher-Langenbeck approach. When facing acetabular fractures, fracture reduction remains the most challenging step [4, 8]. Traditional surgical methods often rely on the surgeon’s experience for fracture reduction and fixation, where conventional plates are manually contoured and shaped to fix the reduced fracture. In recent years, computer simulation surgery and 3D printing technology have been applied to many complex types of fractures, including acetabular fractures. Through 3D-printed physical fracture models, surgeons can better understand the fracture type and determine the optimal reduction sequence, ultimately achieving a strong fixation of the fracture [11, 12]. Huang et al. [13] applied 3D printing technology in the teaching of acetabular fractures, and the results indicated that 3D printed models were beneficial in promoting morphological understanding and subjective interest in acetabular fractures. This advantage arises not only from the three-dimensional display of the fracture morphology but also from the tactile feedback of the real fracture. Currently, the mainstream approach for using 3D printing technology in the treatment of acetabular fractures involves pre-bending and shaping plates based on the fracture model or virtually reduced fracture model, and then using the pre-bent plates for fixation during surgery. Ansari et al. [14] applied traditional surgical methods and 3D printing technology with pre-bent plates in 27 cases of complex acetabular fractures, and the results showed that 3D printing technology helped to better understand the anatomical structure of acetabular fractures, resulting in reduced surgical time, intraoperative blood loss, and fluoroscopy procedures. Chen et al. [15] utilized computer-assisted virtual surgery and 3D printing technology in the treatment of bilateral acetabular column fractures, utilizing fracture models for specific pre-bending of plates, simplifying the surgical process, reducing surgical time, and minimizing invasiveness.

However, for acetabular fractures, even when using 3D printed models for pre-bending plates, achieving ideal anatomical alignment between the plate and the bone cortex can still be challenging [16]. In some cases, it may be necessary to further bend the plates during surgery for proper fixation. In our study, based on the virtually reduced fracture model, the customized plates were designed by the engineers after confirming the placement of the plates and the direction of the screws by the orthopedic surgeons. The fit of the customized plates to the fracture site was then confirmed through computer simulation, and the final product was a completely customized plate that precisely fit the fracture site. The computer-assisted virtual reduction of acetabular fractures is currently performed in two main ways. One approach involves converting the healthy side of the pelvis into the reduced model due to the symmetry of the pelvic skeleton [8, 16, 17]. The other approach involves virtual separation and reduction of the fracture using Mimics software to establish the model after reduction [7, 18]. In our study, we utilized the latter method, which allowed for a better understanding of the shape of the actual fracture fragments. This approach proved effective even in cases of bilateral fractures [2, 6, 19], resulting in a more accurate fit of the customized plates to the patient’s specific anatomy.

With the advancement of 3D printing technology, the concept of individualized custom-made metal plates for the treatment of acetabular fractures has garnered great attention among researchers and the demand for such plates is steadily increasing. Merema et al. [20] applied patient-specific plates and drill guides in the treatment of a complex acetabular fracture, demonstrating the feasibility and potential of this technology in surgical treatment. Robinson et al. [21] studied five cadaveric specimens with acetabular fractures and designed and manufactured corresponding customized fracture plates. The results suggested that customized plates may not be suitable for severe acetabular fractures with more than five fracture fragments. Tomazevic et al. [22] used standardized plastic acetabular bone models to produce individualized custom-made metal plates, demonstrating that customized plates facilitated accurate reduction and fixation of acetabular fractures. However, these studies are currently limited to individual cases or experimental research using cadaveric and plastic bone models, and have not been applied in clinical practice or compared with the same type of acetabular fracture.

In our study, the surgical time was significantly shorter in the 3D group compared to the traditional group (110.0 ± 12.6 vs. 166.0 ± 6.4 min, *p* < 0.001). This can be attributed to the better visualization of fracture reduction based on the 3D models, which significantly reduced the number of intraoperative fluoroscopy procedures (6 ± 2 vs. 8 ± 2, *p* = 0.001). Additionally, the direct use of customized plates in the 3D group reduced the time spent shaping plates during surgery, leading to a significantly lower instrument operation time (34.2 ± 6.2 vs. 58.1 ± 8.6 min, *p* < 0.001). The intraoperative blood loss was also significantly lower in the 3D group (254.9 ± 57.2 vs. 401.6 ± 46.6 mL, *p* < 0.001). These results are consistent with many other studies using pre-bent plates in acetabular fracture surgeries [17, 18, 23, 24]. However, the study by Bouabdellah et al. [8] showed no significant differences in surgical time and intraoperative blood loss between the two methods. The potential reason for this disparity may be associated with the utilization of individualized custom-made metal plates in our study. However, there has not been a comparative study employing customized plates versus pre-bent plates using 3D printing technology. This could be a direction for more comprehensive research in the future for our research group.

In our study, the process of computer-assisted virtual reduction of acetabular posterior column fractures and 3D printing of the physical models took an average of 15.9 ± 3.5 h. The design and production of individualized custom-made metal plates took an average of 32.9 ± 6.3 h. Clinically, patients with acetabular fractures are often in a state of trauma stress, and early surgery may lead to increased intraoperative blood loss and venous thrombosis due to the patient’s hypercoagulable state. Therefore, patients with acetabular and pelvic fractures are usually recommended for surgery 4 to 10 days after the injury [25, 26]. There were no significant differences in hospitalization time and time from injury to surgery between the two groups (*p* = 0.276 and *p* = 0.146, respectively), and the production of 3D printed models and individualized custom-made metal plates did not result in any significant time delays.

In this study, although the healing time of fractures in the 3D printing group was numerically lower than that in the traditional group, no statistically significant difference was observed (*p* = 0.215). This result may be associated with the rich blood supply in the posterior region of the acetabulum, suggesting a potentially crucial role of this blood supply in the process of fracture healing. It is noteworthy that the 3D printing group exhibited higher numerical values in postoperative fracture reduction quality and 6-month Harris scores compared to the traditional group, but these differences did not reach statistical significance (*p* = 0.333 and *p* = 0.394, respectively). The 3D printing group did not demonstrate a significant advantage over the traditional group in these two indicators. We consider that this may be attributed to the critical role played by the surgical team’s reduction techniques or the relatively small sample size in this study. However, in terms of the 12-month Harris score and the 12-month NRS score, the 3D printing group showed significantly better performance than the traditional group, with statistically significant differences (*p* = 0.037 and *p* = 0.017, respectively). This suggests that the combination of 3D printing technology with individualized custom-made metal plates can significantly improve the long-term recovery quality of hip joint function. There was no significant difference in complications between the two groups of patients (*p* = 0.433). It is noteworthy that the complications in the 3D group were unrelated to the 3D printing technology and individualized custom-made metal plates.

This study has some limitations. First, it is a retrospective and non-randomized study. Second, the measurement of postoperative fracture reduction quality may be more accurate on CT scans than on plain X-rays. Finally, due to the rarity of posterior column acetabular fractures, the sample size in this study is relatively small, and future studies with larger sample sizes are needed to further validate the effectiveness of 3D printing technology combined with individualized custom-made metal plates in the treatment of posterior column acetabular fractures.

## Conclusion

Combining 3D printing with individualized custom-made metal plates for acetabular posterior wall and column fractures reduces surgery and instrument time, minimizes intraoperative procedures and blood loss, enhancing long-term hip joint function recovery.

### Electronic supplementary material

Below is the link to the electronic supplementary material.

Supplementary Material 1

## Data Availability

Due to ethical approval restrictions involving patient data and anonymity, the data in this study will not be publicly released but can be made available upon reasonable request from the corresponding author.

## Declarations

### Conficts of interest

The authors declare that they have no conficts of interest concerning this article.
