# Supplementary material for: Surgical treatment outcomes of acetabular posterior wall and posterior column fractures using 3D printing technology and individualized custom-made metal plates: a retrospective study
Source: BMC Surg. 2024 May 16;24:157. doi: 10.1186/s12893-024-02451-x (PMC11097422; doi:10.1186/s12893-024-02451-x)
Supplement: Supplementary file 1 — Supplementary Material 1 [file 12893_2024_2451_MOESM1_ESM.docx]

**Anonymous individual patient data**

| Case | gruop | Gender (M/F) | Age (years) | BMI (kg/m²) | Surgical Time (minutes) | Instrument Operation Time (minutes) | Intraoperative Blood Loss (mL) | X-ray Fluoroscopy (frequency) |
| --- | --- | --- | --- | --- | --- | --- | --- | --- |
| 1 | 3D Printing Group | F | 35 | 24.4 | 105.5 | 35.6 | 254 | 6 |
| 2 | 3D Printing Group | M | 23 | 22.3 | 121.5 | 40.1 | 184 | 5 |
| 3 | 3D Printing Group | F | 45 | 19.8 | 100.6 | 33.5 | 212 | 7 |
| 4 | 3D Printing Group | M | 38 | 27.9 | 110.5 | 30.3 | 290 | 6 |
| 5 | 3D Printing Group | M | 27 | 29.1 | 135.4 | 35.6 | 350 | 5 |
| 6 | 3D Printing Group | M | 51 | 23.4 | 140.5 | 40.1 | 400 | 9 |
| 7 | 3D Printing Group | M | 37 | 30.8 | 110.5 | 30.4 | 263 | 5 |
| 8 | 3D Printing Group | F | 54 | 25.2 | 105.1 | 25.3 | 254 | 5 |
| 9 | 3D Printing Group | M | 38 | 24.9 | 100.5 | 22.5 | 286 | 6 |
| 10 | 3D Printing Group | M | 39 | 25.7 | 95.8 | 28.5 | 189 | 4 |
| 11 | 3D Printing Group | M | 25 | 22.6 | 98.6 | 33.5 | 196 | 5 |
| 12 | 3D Printing Group | M | 24 | 28.1 | 108.5 | 41.5 | 263 | 6 |
| 13 | 3D Printing Group | M | 41 | 25.7 | 100.2 | 45.4 | 241 | 10 |
| 14 | 3D Printing Group | M | 27 | 25.1 | 107.4 | 34.5 | 232 | 6 |
| 15 | 3D Printing Group | M | 28 | 19.4 | 108.9 | 40.3 | 221 | 5 |
| 16 | 3D Printing Group | M | 31 | 25.3 | 109.8 | 30.4 | 243 | 7 |
| 17 | Traditional Group | M | 42 | 24.3 | 169.6 | 72.1 | 383 | 9 |
| 18 | Traditional Group | M | 51 | 19.6 | 158.8 | 62.6 | 391 | 10 |
| 19 | Traditional Group | F | 28 | 26.8 | 160.5 | 52.1 | 510 | 9 |
| 20 | Traditional Group | F | 26 | 21.4 | 170.6 | 62.5 | 421 | 12 |
| 21 | Traditional Group | M | 22 | 25.7 | 168.8 | 70.6 | 281 | 10 |
| 22 | Traditional Group | M | 46 | 30.1 | 166.4 | 61.1 | 410 | 8 |
| 23 | Traditional Group | F | 38 | 23.6 | 165.6 | 55.8 | 380 | 7 |
| 24 | Traditional Group | M | 35 | 22.6 | 172.1 | 45.5 | 391 | 8 |
| 25 | Traditional Group | M | 37 | 25.7 | 167.8 | 52.2 | 385 | 10 |
| 26 | Traditional Group | M | 42 | 24.8 | 174.6 | 49.5 | 421 | 6 |
| 27 | Traditional Group | M | 59 | 25.3 | 166.7 | 59.8 | 411 | 6 |
| 28 | Traditional Group | F | 28 | 25.2 | 155.4 | 72.1 | 412 | 7 |
| 29 | Traditional Group | M | 25 | 25.1 | 170.5 | 49.5 | 420 | 9 |
| 30 | Traditional Group | M | 31 | 22.5 | 152.6 | 50.5 | 430 | 7 |
| 31 | Traditional Group | F | 49 | 24.4 | 169.7 | 55.6 | 378 | 6 |

| Case | gruop | Fracture Reduction Quality | | 6-month Harris Score | 12-month Harris Score | Complications |
| --- | --- | --- | --- | --- | --- | --- |
| 1 | 3D Printing Group | good | 85 | | 86 | N |
| 2 | 3D Printing Group | good | 90 | | 87 | N |
| 3 | 3D Printing Group | good | 88 | | 89 | N |
| 4 | 3D Printing Group | good | 85 | | 91 | N |
| 5 | 3D Printing Group | good | 90 | | 95 | N |
| 6 | 3D Printing Group | Fair | 71 | | 77 | heterotopic ossification |
| 7 | 3D Printing Group | good | 81 | | 82 | inflammatory reactions |
| 8 | 3D Printing Group | good | 87 | | 85 | N |
| 9 | 3D Printing Group | good | 92 | | 89 | N |
| 10 | 3D Printing Group | good | 88 | | 87 | N |
| 11 | 3D Printing Group | good | 83 | | 85 | N |
| 12 | 3D Printing Group | good | 85 | | 86 | N |
| 13 | 3D Printing Group | good | 75 | | 81 | inflammatory reactions |
| 14 | 3D Printing Group | good | 89 | | 85 | N |
| 15 | 3D Printing Group | good | 85 | | 86 | N |
| 16 | 3D Printing Group | good | 82 | | 84 | N |
| 17 | Traditional Group | good | 84 | | 82 | N |
| 18 | Traditional Group | good | 85 | | 87 | N |
| 19 | Traditional Group | good | 82 | | 85 | N |
| 20 | Traditional Group | Fair | 81 | | 75 | inflammatory reactions |
| 21 | Traditional Group | Fair | 70 | | 72 | iatrogenic nerve symptoms |
| 22 | Traditional Group | good | 84 | | 83 | N |
| 23 | Traditional Group | good | 78 | | 75 | N |
| 24 | Traditional Group | good | 87 | | 89 | N |
| 25 | Traditional Group | good | 85 | | 88 | N |
| 26 | Traditional Group | good | 86 | | 85 | N |
| 27 | Traditional Group | good | 81 | | 74 | inflammatory reactions |
| 28 | Traditional Group | Fair | 70 | | 73 | heterotopic ossification |
| 29 | Traditional Group | good | 86 | | 86 | N |
| 30 | Traditional Group | good | 73 | | 71 | traumatic arthritis |
| 31 | Traditional Group | good | 90 | | 91 | N |
